# Primary Lung Squamous Cell Carcinoma With Intestinal Metastasis: A Case Report and Literature Review

**DOI:** 10.1111/crj.13817

**Published:** 2024-08-08

**Authors:** Jin Tao, Zhiqiang Wu, Rongfei Huang, Jun Li, Tiewei Xu, Yujie Gao, Weikun Jia, Hu Chen

**Affiliations:** ^1^ Department of Cardiothoracic Surgery School of Clinical Medicine and The First Affiliated Hospital of Chengdu Medical College Chengdu China; ^2^ Department of Pathology School of Clinical Medicine and The First Affiliated Hospital of Chengdu Medical College Chengdu China; ^3^ Department of Gastroenterology School of Clinical Medicine and The First Affiliated Hospital of Chengdu Medical College Chengdu China; ^4^ Department of Stomatology School of Clinical Medicine and The First Affiliated Hospital of Chengdu Medical College Chengdu China

**Keywords:** case reports, intestinal metastasis, lung cancer, lung squamous cell carcinoma (LUSC), metastasis

## Abstract

Lung squamous cell carcinoma (LUSC) is characterized by a high rate of metastasis and recurrence, leading to a poor prognosis for affected patients. Intestinal metastasis of LUSC is a rare clinical occurrence. Treatment options for LUSC patients with intestinal metastasis are limited, and no standard therapy guidelines exist for managing these cases. In this review, we discuss the clinical features, diagnosis, and treatment of LUSC patients with intestinal metastasis and present a rare case of LUSC with intestinal metastasis. We describe a patient who presented with a severe cough and chest pain and diagnosed with LUSC and bone tumor. Initially, the primary LUSC and bone tumor were controlled with standard treatments. However, the primary LUSC reoccurred shortly after treatment, this time with intestinal metastasis, for which effective treatments are lacking. Our observation from the case suggests that LUSC metastasizing to intestinal tract is associated with a poorer prognosis.

## Graphical abstract

Lung squamous cell carcinoma (LUSC), accounting for 20%–30% of non‐small cell lung cancer (NSCLC) cases, is associated with high mortality. In its early stages, the symptoms of LUSC are mild and often go unnoticed. Consequently, most patients are diagnosed at an advanced stage. Intestinal metastasis from lung cancer is a relatively rare occurrence. Clinically, diagnosing intestinal metastasis of LUSC primarily relies on imaging examination results. Here, we review the clinical features, diagnosis, and treatment of LUSC patients with intestinal metastasis and report a rare case of LUSC accompanied by intestinal metastasis.

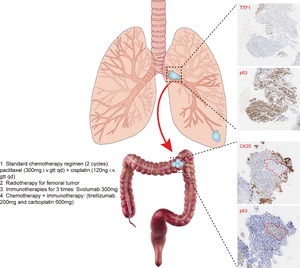

## 1 Introduction

Lung squamous cell carcinoma (LUSC), accounting for 20%–30% of non‐small cell lung cancer (NSCLC) cases, is associated with high mortality rates [1]. In its early stages, LUSC symptoms are mild and are often go unnoticed. Consequently, most patients are diagnosed at an advanced stage [2]. Intestinal metastasis from lung cancer is a relatively rare, and its clinical diagnosis primarily relies on imaging examinations [3]. In this review, we discuss the clinical features, diagnosis, and treatment of LUSC patients with intestinal metastasis and present a rare case of LUSC accompanied by intestinal metastasis.

## 2 Case Presentation

**FIGURE 1:**
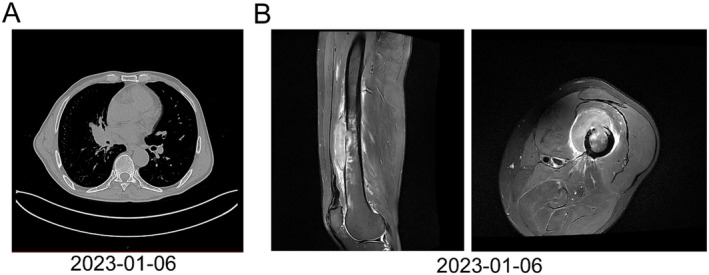
Enhanced computed tomography (CT) scans. (A) Chest scans indicated the presence of a right hilum nodule in the lung. (B) Leg scans showed tumor infiltration in the femur.

**FIGURE 2:**
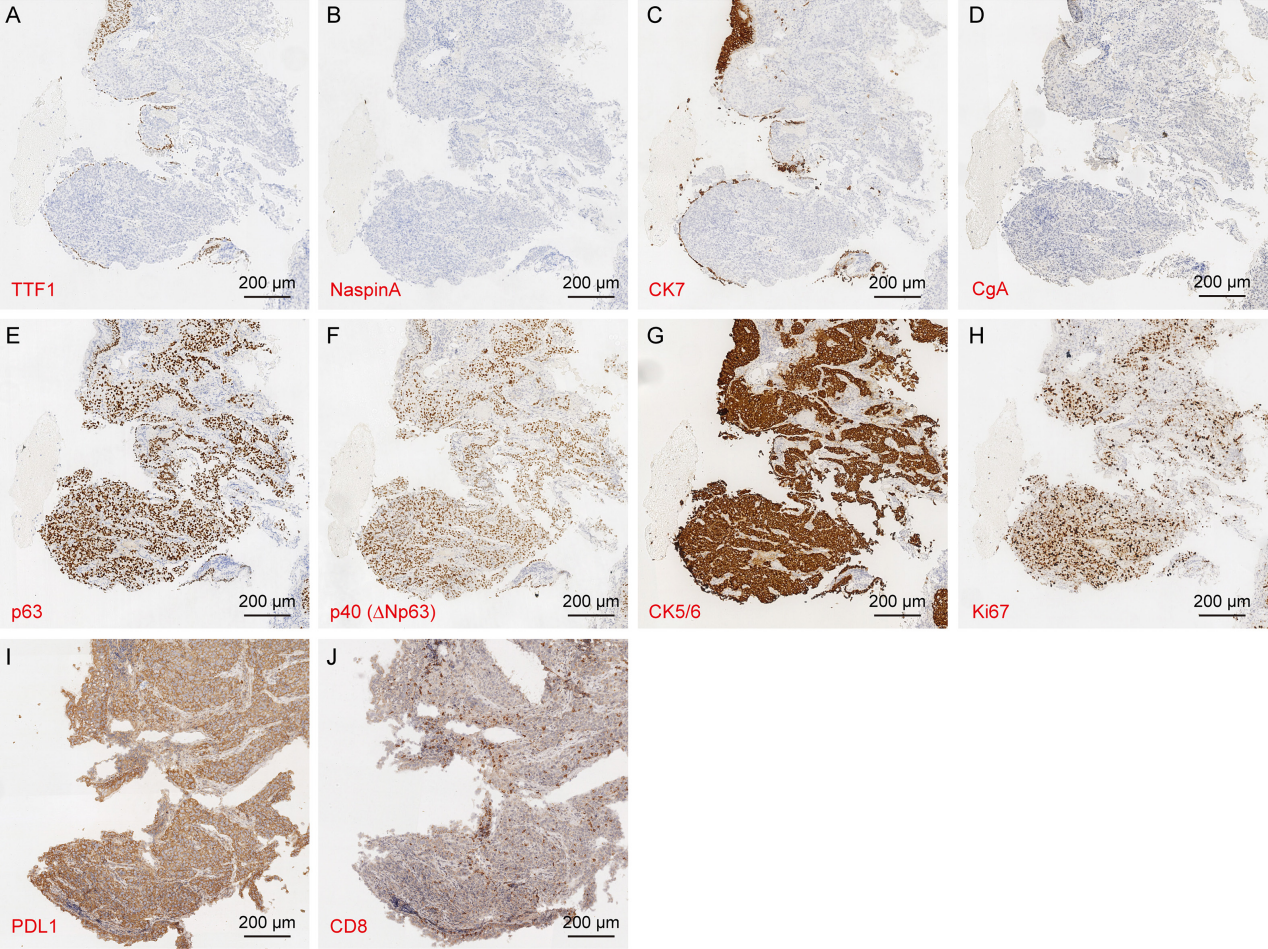
Immunohistochemical staining of primary squamous cell carcinoma for TTF1 (A), NaspinA (B), CK7 (C), CgA (D), p63 (E), p40 (F), CK5/6 (G), Ki67 (H), PDL1 (I), and CD8 (J).

A 55‐year‐old male presented with a severe cough accompanied by chest pain and was diagnosed with lung cancer in the right hilum of the lung (Figure 1A) and a tumor in the left femur (Figure 1B) at our hospital on January 6, 2023. The patient had a history of smoking, beginning at Age 10, with consumption gradually increasing from 10 to 20 cigarettes per day until he quit smoking in June 2022. He also began drinking alcohol at the age of 21, with intake progressively increasing to 350 mL (52%, v/v) per day, and ceased alcohol consumption in December 2022. Between 1997 and 2004, he worked in a coal mine, where he was chronically exposed to dust and other toxic and harmful gases. In 2003, he started experiencing symptoms of a dry cough without expectoration or hemoptysis, which exacerbated annually for at least 3 months each year. Despite these symptoms, he had never sought medical attention or treatment prior to the lung cancer diagnosis. A pathological biopsy confirmed the primary lung cancer as poorly differentiated LUSC with the following immunohistochemical profile: TTF1 (−), NaspinA (−), CK7 (−), CgA (−), p63 (−), p40 (+), p63 (+), CK5/6 (+), and Ki67 (+) (Figure 2). We administered the standard chemotherapy regimen for primary squamous lung cancer: paclitaxel (300 mg i.v.gtt qd) combined with cisplatin (120 ng i.v. gtt qd). After two‐cycle chemotherapies on January 17, 2023, and February 7, 2023, the patient's cough symptoms were alleviated, and the primary lesion significantly reduced in size. Due to femoral pain caused by the femoral tumor, the patient received radiotherapy in the Oncology Department of our hospital on March 17, 21, and 27, 2023.

**FIGURE 3:**
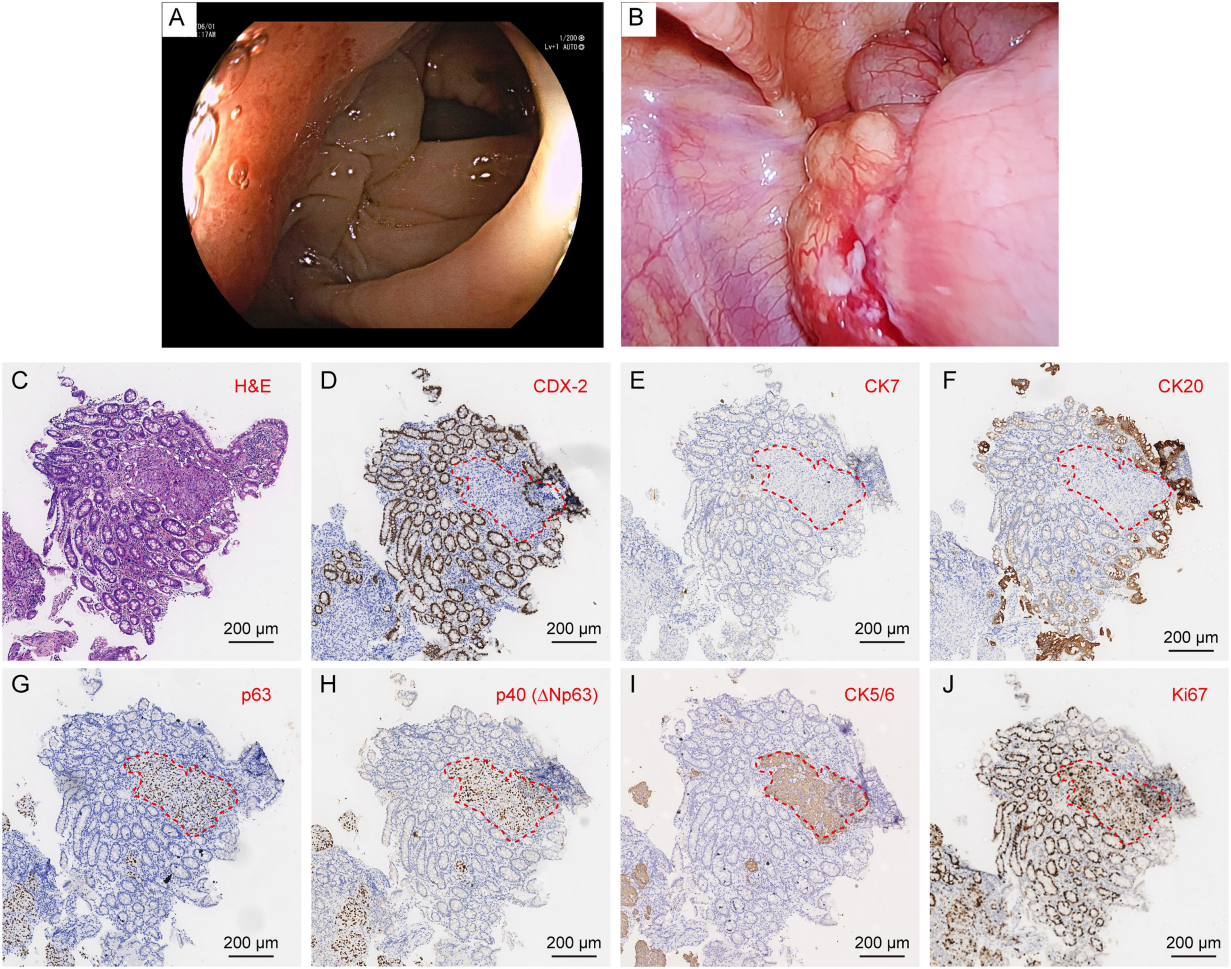
The images of tumor on the colon. (A) Colonoscopy showed nodular eminence in transverse colon. (B) Laparoscopy revealed a significant tumor mass on the colon. (C) Hematoxylin‐eosin staining of colonic tumor. (F–J) Immunohistochemical staining of CDX‐2 (D), CK7 (E), CK20 (F), p63 (G), p40 (H), CK5/6 (I), and Ki67 (J).

During radiotherapy, we detected the expression of PDL1 and CD8 in LUSC samples using immunohistochemistry. We found that almost all LUSC cells expressed PDL1, while only a few cells expressed CD8 (Figure 2I,J). Consequently, we administered immune checkpoint inhibitors to the patient three times: Svolumab 300 mg (March 8, 2023), Svolumab 120 mg (March 16, 2023), and Svolumab 300 mg (March 29, 2023). Following these treatments, the patient's femoral pain was alleviated. However, a subsequent chest CT scan revealed that the primary lung tumor had increased in size compared to the scan from January 6, 2023. We recommend chemotherapy combined with immunotherapy (tirellizumab 200 mg and carboplatin 600 mg) on April 21, 2023. Unexpectedly, on June 1, 2023, the patient experienced repeated difficulty with defecation. Colonoscopy and laparoscopy revealed nodular eminence in transverse colon (Figure 3A,B). Pathological results confirmed squamous cell carcinoma, likely originating from the lung: CDX‐2 (−), CK7 (−), CK20 (−), p63 (+), p40 (+), CK5/6 (+), and Ki67 (+) (Figure 3C–J). To address the defection issues, a transverse colostomy was performed in the Gastroenterostomy Department of our hospital on June 30, 2023. Extensive abdominal malignant tumor metastasis was discovered during the operation. The surgery was uneventful, and the patient experienced relief from postoperative abdominal pain. However, due to reliance solely on parenteral nutrition postsurgery, the patient's vital signs deteriorated, and he developed cachexia. One week later, the patient's abdominal pain recurred, and he developed nausea and vomiting after eating. He was transferred to the Oncology Department for further treatment. Over the following month, the patient experienced upper gastrointestinal bleeding and jaundice. Imaging studies indicated extensive tumor progression, and the patient entered the cachectic phase of malignant tumors. Palliative care was planned, and after discussions with the patient and his family, he refused in‐hospital palliative treatment and requested to be discharged. No further treatment was administered subsequently.

## 3 Discussion

The probability of gastrointestinal metastasis from lung cancer varies from 4.7% to 14% [4, 5, 6, 7, 8, 9, 10, 11, 12, 13, 14, 15]. This reports a rare case of LUSC with metastasis to both intestines and bones. Unfortunately, the patient had bone metastases and was not a candidate for surgery at the time of diagnosis. Consequently, we relied on immunotherapy, chemotherapy, and radiotherapy to manage the condition and alleviate pain. In this study, we administered the standard chemotherapy regimen for primary LUSC, supplemented with immunotherapy and radiotherapy for bone tumors. Following these treatments, the disease was controlled to some extent, and the patient's quality of life improved. However, the primary lesion soon became uncontrolled again, now accompanied by intestinal metastasis. This development suggests that LUSC patients with intestinal metastasis have a poorer prognosis when treated with standard therapy regimens [16, 17]. Therefore, it is imperative to explore more therapeutic methods and effective regimens urgently.

Unlike LUAD, LUSC lacks effective molecular targets, making targeted therapies rare for LUSC [18, 19, 20]. Differentiating LUSC from LUAD is crucial for selecting appropriate drug during lung cancer treatment. However, treatment options for first‐line therapy of advanced LUSC remain limited compared to other types of lung cancer. Patients with advanced LUSC primarily benefit from radiotherapy, chemotherapy, or immunotherapy. Platinum‐based chemotherapies are the main regimens for advanced LUSC and can effectively improve overall survival. Unfortunately, rare cases with intestinal metastasis have a worse outcome, with an overall survival of only 4–8 weeks [16, 17, 21]. Table 1 summarizes previous reports on therapies for intestinal metastasis of LUSC. All patients received radiotherapy, chemotherapy, or surgery for gastrointestinal tumors after the onset of gastrointestinal symptoms, but no standard treatment exists for LUSC with gastrointestinal metastasis. Most cases involved symptomatic treatment to improve quality of life and relatively prolong survival time. Recently, some clinical trials (RATIONALE 307 and ORIENT‐12) have shown that patients with advanced squamous NSCLC benefit from immunotherapies. However, in this case, the patient's symptoms did not significantly improve after immunotherapy, indicating that further development and improvement of immunotherapy are needed.

**TABLE 1:** The previously reported lung squamous cell carcinoma with intestinal metastasis.

Author (year)	Age/sex	Location of lung cancer	Transfer site	Treatment	Survival after treatment	Reference PMID
Smith and Vlasak (1978)	57/M	Left lower lobe	Sigmoid colon	Unknown	1 year	669 161
Coffman et al. (1980)	63/M	Left upper lobe	Sigmoid colon	Unknown	Unknown	6 336 970
Wegener et al. (1988)	69/M	Right medial lobe	Ascending colon	Chemoradiotherapy + surgical treatment	2 months	2 851 891
Bastos et al. (1998)	69/M	Right main bronchus	Sigmoid colon	Unknown	2 months	9 649 029
Carroll and Rajesh (2001)	68/M	Right lower lobe of lung	Sigmoid colon	Unknown	6 months	11 343 961
John, Kotru, and Pearson (2002)	73/M	Right lower lobe	Entire colon	Untreated	3 months	12 432 195
Uner et al. (2005)	58/M	Left lower lung lobe	Descending colon	Left lower lobectomy and left hemicolectomy	Alive before submission	15 875 637
Hirasaki et al. (2008)	74/M	Light upper lobe	Descending colon	Unknown	8 months	18 803 365
Lau and Leung (2008)	59/M	Left hilar mass	Caecum	Chemoradiotherapy	Unknown	18 382 025
Meneses Grasa et al. (2009)	69/M	Left upper lobe of lung	Mesentery	Surgery + chemotherapy	1.5 months	20 001 163
Yamada et al. (2011)	66/M	Left lung field	Lumen of the third portion of the duodenum	Chemoradiotherapy	Alive before submission	22 026 313
Sakai et al. (2012)	60/M	Right upper lobe of lung	Colon	Surgery + chemotherapy	6 months	22 741 562
Lou et al. (2014)	64/M	Right upper lobe of lung	Colon	Surgery + chemotherapy	5 months	24 914 356
Miyazaki et al. (2015)	54/M	Right upper lobe of lung	Stomach, colon, caecum	Surgery + chemotherapy	Unknown	25 491 474
Liu et al. (2015)	66/M	Right lower lobe of lung	Small intestine	Surgery + chemotherapy	Alive before submission	25 805 957
Li et al. (2018)	61/M	Right upper lobe of lung	Stomach, small intestine	Chemoradiotherapy	7 months	29 901 596
Tanaka et al. (2019)	78/M	Right upper lobe	Splenic	Splenectomy and right upper lobectomy separately	15 months	31 845 086
He et al. (2019)	61/M	Left lower lung lobe	Large gastric stromal	Left lower lobe resection, cardia resection with anastomosis of the esophagus and stomach below the aortic arch, and lymphadenectomy/chemoradiotherapy	Alive before submission	30 561 123
Nemoto et al. (2020)	64/M	Right lower lobe	Gastric fundal	Gastrectomy	1 year	32 212 371
Chandra et al. (2020)	69/M	Right lung	Sigmoid, liver	Sigmoidoscopy with end colostomy, partial omentectomy, and wedge resection of Liver Segment III were performed	Unknown	32 963 763
Zhu et al. (2022)	65/M	Left lung	Jejunal	ESD + chemoradiotherapy + immunotherapy	Alive before submission	35 571 658
Li et al. (2023)	50/M	Left lower lung lobe	Appendix, cecum, top cranial skin, lumbar vertebra, inguinal groin, and left ventricle	Chemoradiotherapy	4 months	37 152 036

Abbreviations: F, female; M, male.

## Author Contributions

**Jin Tao:** study concept or design and data collection. **Zhiqiang Wu:** data analysis or interpretation and writing the paper. **Rongfei Huang** and **Jun Li:** data collection. **Tiewei Xu** and **Yujie Gao:** data analysis or interpretation. **Hu Chen** and **Weikun Jia:** study concept or design and writing the paper. All authors contributed to the article and approved the submitted version.

## Ethics Statement

Appropriate written informed consent was obtained from the patient for the publication of this case report and accompanying images. It was approved by the Institutional Review Board of The First Affiliated Hospital of Chengdu Medical College and was implemented.

## Conflicts of Interest

The authors declare no conflicts of interest.

## Data Availability

The data that support the findings of this study are available from the corresponding author upon reasonable request.

## References

[1] Z. Chen , C. M. Fillmore , P. S. Hammerman , C. F. Kim , and K. K. Wong , “Non‐Small‐Cell Lung Cancers: A Heterogeneous Set of Diseases,” Nature Reviews Cancer 14 (2014): 535–546. 25056707 10.1038/nrc3775 PMC5712844

[2] K. D. Miller , L. Nogueira , T. Devasia , et al., “Cancer Treatment and Survivorship Statistics, 2022,” CA: A Cancer Journal for Clinicians 72 (2022): 409–436. 35736631 10.3322/caac.21731

[3] F. Y. Niu , Q. Zhou , J. J. Yang , et al., “Distribution and Prognosis of Uncommon Metastases From Non‐Small Cell Lung Cancer,” BMC Cancer 16 (2016): 149. 26911831 10.1186/s12885-016-2169-5 PMC4766662

[4] P. M. McNeill , L. D. Wagman , and J. P. Neifeld , “Small Bowel Metastases From Primary Carcinoma of the Lung,” Cancer 59 (1987): 1486–1489. 3028602 10.1002/1097-0142(19870415)59:8<1486::aid-cncr2820590815>3.0.co;2-w

[5] H. J. Smith and M. G. Vlasak , “Metastasis to the Colon From Bronchogenic Carcinoma,” Gastrointestinal Radiology 2 (1978): 393–396. 669161 10.1007/BF02256525

[6] K. L. Brown , R. A. Beg , M. A. Demany , and M. A. Lacerna , “Rare Metastasis of Primary Bronchogenic Carcinoma to Sigmoid Colon: Report of a Case,” Diseases of the Colon and Rectum 23 (1980): 343–345. 7398508 10.1007/BF02586842

[7] M. Wegener , G. Börsch , E. Reitemeyer , and K. Schäfer , “Metastasis to the Colon From Primary Bronchogenic Carcinoma Presenting as Occult Gastrointestinal Bleeding‐‐Report of a Case,” Zeitschrift für Gastroenterologie 26 (1988): 358–362. 2851891

[8] D. Carroll and P. B. Rajesh , “Colonic Metastases From Primary Squamous Cell Carcinoma of the Lung,” European Journal of Cardio‐Thoracic Surgery 19 (2001): 719–720. 11343961 10.1016/s1010-7940(01)00646-7

[9] A. K. John , A. Kotru , and H. J. Pearson , “Colonic Metastasis From Bronchogenic Carcinoma Presenting as Pancolitis,” Journal of Postgraduate Medicine 48 (2002): 199–200. 12432195

[10] T. E. Stinchcombe , M. A. Socinski , L. M. Gangarosa , and A. H. Khandani , “Lung Cancer Presenting With a Solitary Colon Metastasis Detected on Positron Emission Tomography Scan,” Journal of Clinical Oncology 24 (2006): 4939–4940. 17050879 10.1200/JCO.2006.06.3354

[11] C. J. Yang , J. J. Hwang , W. Y. Kang , et al., “Gastro‐Intestinal Metastasis of Primary Lung Carcinoma: Clinical Presentations and Outcome,” Lung Cancer 54 (2006): 319–323. 17010474 10.1016/j.lungcan.2006.08.007

[12] S. Hirasaki , S. Suzuki , S. Umemura , H. Kamei , M. Okuda , and K. Kudo , “Asymptomatic Colonic Metastases From Primary Squamous Cell Carcinoma of the Lung With a Positive Fecal Occult Blood Test,” World Journal of Gastroenterology 14 (2008): 5481–5483. 18803365 10.3748/wjg.14.5481 PMC2744902

[13] C. P. Lau and W. K. Leung , “Caecal Metastasis From a Primary Small‐Cell Lung Carcinoma,” Hong Kong Medical Journal 14 (2008): 152–153. 18382025

[14] M. W. Weng , H. C. Wang , J. C. Chiou , S. L. Lin , and R. S. Lai , “Colonic Metastasis From a Primary Adenocarcinoma of the Lung Presenting With Acute Abdominal Pain: A Case Report,” Kaohsiung Journal of Medical Sciences 26 (2010): 40–44. 20040472 10.1016/S1607-551X(10)70007-3 PMC11916347

[15] A. Pezzuto , S. Mariotta , F. Fioretti , and S. Uccini , “Metastasis to the Colon From Lung Cancer Presenting With Severe Hyponatremia and Dyspnea in a Young Male: A Case Report and Review of the Literature,” Oncology Letters 5 (2013): 1477–1480. 23761813 10.3892/ol.2013.1208 PMC3678879

[16] Y. Hu , N. Feit , Y. Huang , W. Xu , S. Zheng , and X. Li , “Gastrointestinal Metastasis of Primary Lung Cancer: An Analysis of 366 Cases,” Oncology Letters 15 (2018): 9766–9776. 29928351 10.3892/ol.2018.8575 PMC6004691

[17] N. Taira , T. Kawabata , A. Gabe , et al., “Analysis of Gastrointestinal Metastasis of Primary Lung Cancer: Clinical Characteristics and Prognosis,” Oncology Letters 14 (2017): 2399–2404. 28781676 10.3892/ol.2017.6382 PMC5530210

[18] K. König , M. Peifer , J. Fassunke , et al., “Implementation of Amplicon Parallel Sequencing Leads to Improvement of Diagnosis and Therapy of Lung Cancer Patients,” Journal of Thoracic Oncology 10 (2015): 1049–1057. 26102443 10.1097/JTO.0000000000000570

[19] A. Caliò , A. Nottegar , E. Gilioli , et al., “ALK/EML4 Fusion Gene May Be Found in Pure Squamous Carcinoma of the Lung,” Journal of Thoracic Oncology 9 (2014): 729–732. 24722159 10.1097/JTO.0000000000000109

[20] Y. Miyamae , K. Shimizu , J. Hirato , et al., “Significance of Epidermal Growth Factor Receptor Gene Mutations in Squamous Cell Lung Carcinoma,” Oncology Reports 25 (2011): 921–928. 21318227 10.3892/or.2011.1182

[21] S. Cedrés , N. Mulet‐Margalef , M. A. Montero , P. Martinez , A. Martínez , and E. Felip , “Rectal Metastases From Squamous Cell Carcinoma: A Case Report and Review of the Literature,” Case Reports in Medicine 2012 (2012): 947524. 22567021 10.1155/2012/947524 PMC3332391

